# Thrombus stiffness depends on occlusion site and influences thrombectomy outcomes in acute ischemic stroke

**DOI:** 10.1093/esj/aakag132

**Published:** 2026-11-09

**Authors:** Luca Bontempi, Janneke M H Cruts, Rachel M E Cahalane, Otilia E Ilie, Nikki Boodt, Daniel Bos, Behrooz Fereidoonnezhad, Pieter Jan van Doormaal, Aad van der Lugt, Diederik W J Dippel, Frank J H Gijsen

**Affiliations:** Department of Biomedical Engineering, Thoraxcenter, Erasmus MC, University Medical Center, Rotterdam, The Netherlands; Department of Radiology and Nuclear Medicine, Erasmus MC, University Medical Center, Rotterdam, The Netherlands; Department of Biomechanical Engineering, Delft University of Technology, Delft, The Netherlands; Department of Biomedical Engineering, Thoraxcenter, Erasmus MC, University Medical Center, Rotterdam, The Netherlands; Department of Biomedical Engineering, Thoraxcenter, Erasmus MC, University Medical Center, Rotterdam, The Netherlands; School of Engineering, Bernal Institute, University of Limerick, Limerick, Ireland; Department of Biomedical Engineering, Thoraxcenter, Erasmus MC, University Medical Center, Rotterdam, The Netherlands; Department of Radiology and Nuclear Medicine, Erasmus MC, University Medical Center, Rotterdam, The Netherlands; Department of Neurology, Erasmus MC, University Medical Center, Rotterdam, The Netherlands; Department of Public Health, Erasmus MC, University Medical Center, Rotterdam, The Netherlands; Department of Radiology and Nuclear Medicine, Erasmus MC, University Medical Center, Rotterdam, The Netherlands; Department of Epidemiology, Erasmus MC, University Medical Center, Rotterdam, The Netherlands; Department of Biomechanical Engineering, Delft University of Technology, Delft, The Netherlands; Department of Radiology and Nuclear Medicine, Erasmus MC, University Medical Center, Rotterdam, The Netherlands; Department of Radiology and Nuclear Medicine, Erasmus MC, University Medical Center, Rotterdam, The Netherlands; Department of Neurology, Erasmus MC, University Medical Center, Rotterdam, The Netherlands; Department of Biomedical Engineering, Thoraxcenter, Erasmus MC, University Medical Center, Rotterdam, The Netherlands; Department of Biomechanical Engineering, Delft University of Technology, Delft, The Netherlands

**Keywords:** acute ischemic stroke, endovascular thrombectomy, thromboemboli, blood clot, thrombus mechanics, thrombus histology, CT imaging, angiographic outcomes

## Abstract

**Introduction:**

Only 60% of patients with acute ischemic stroke (AIS) achieve successful reperfusion after the first endovascular thrombectomy (EVT) attempt. *In vitro* studies have shown that thrombus stiffness influences thrombus-device interaction and retrieval efficacy. This prospective observational study investigates how thrombus stiffness relates to clinical factors, thrombus imaging characteristics and histological composition. It further explores the association between thrombus stiffness and EVT success, as measured by the modified first-pass reperfusion (mFPR).

**Patients and methods:**

A total of 281 thrombus samples were collected from 51 AIS patients undergoing EVT. Thrombus stiffness was quantified using compressive mechanical testing, composition from histological staining and radiological characteristics from pre-interventional non-contrast computed tomography (CT) and CT angiography. Statistical analysis was performed using linear and logistic mixed-effects regression models.

**Results:**

Thrombi retrieved from the proximal M2 segment of the middle cerebral artery showed significant lower stiffness (0.236 kPa, 95% CI, 0.136–0.409) compared to those from the M1 segment (0.760 kPa, 95% CI, 0.492–1.174) and from the ICA (1.106 kPa, 95% CI, 0.558–2.192). Occlusion location was the strongest clinical marker of thrombus stiffness. Adjusted mFPR probability was 40.4% (95% CI, 24.5–67.0) for thrombi retrieved from the ICA, 89.7% (95% CI, 79.5–97.0) for M1 occlusions, and 53.0% (95% CI, 30.8–84.7) for M2 occlusions.

**Conclusion:**

Thrombi in distal vessels of the anterior cerebral circulation are significantly softer than those in proximal intracranial arteries. First-pass EVT success is lower for the stiffest thrombi from the ICA and for the softest thrombi from M2, providing a foundation for future biomechanically informed thrombus removal strategies to optimise AIS treatment.

## Graphical abstract

**Graphical abstract:**
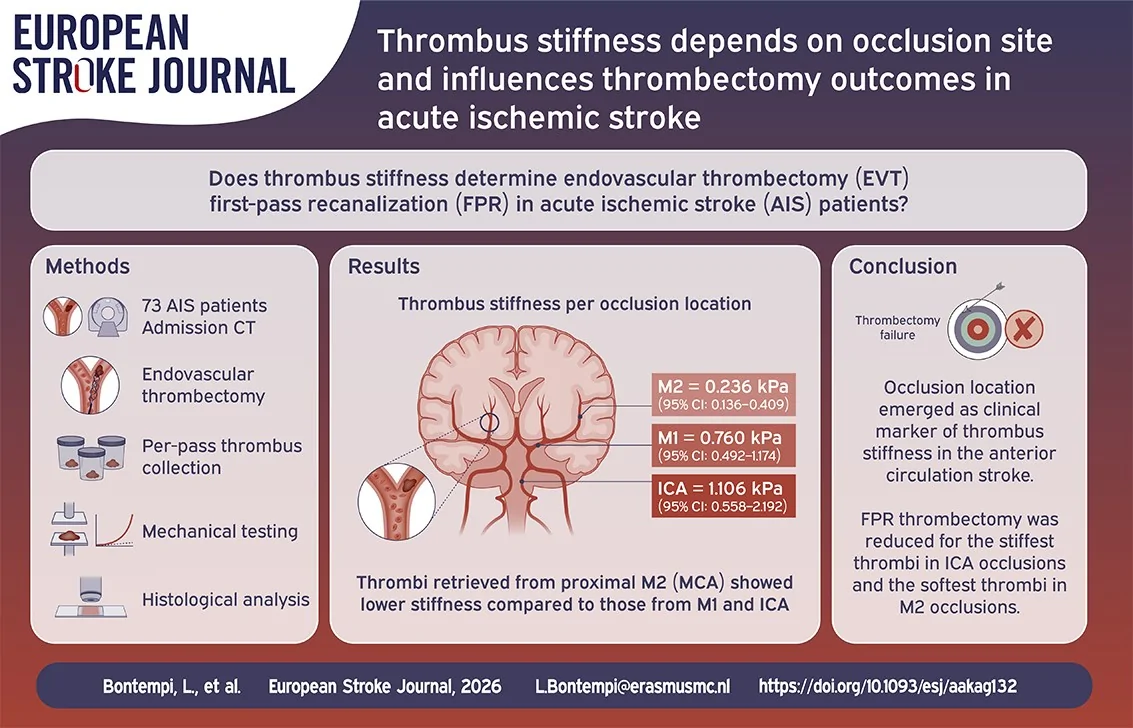

## Teaser

**Previous literature**
 

- Thrombi retrieved from acute ischemic stroke patients show substantial inter- and intra-patient variability in composition, mechanical properties and radiological characteristics, which is not adequately represented in blood clot analogue studies.
- Thrombus mechanical properties and their interaction with the thrombectomy devices may provide valuable insights as potential determinants of procedural success in acute ischemic stroke interventions.

**Key findings**
 

- Thrombi in distal vessels are significantly softer than those in proximal intracranial arteries.
- First evaluation of the association between thrombus stiffness and EVT success, showing that first-pass thrombectomy success is reduced for the stiffest thrombi in ICA occlusions and the softest thrombi in M2 occlusions.

**Knowledge advancement**
 

▪ Occlusion location emerged as clinical marker of thrombus stiffness in the anterior circulation stroke.
▪ Thrombectomy device design and procedural strategy may benefit from considering thrombus mechanical properties differences across occlusion sites.

## Introduction

Despite the proven efficacy of endovascular thrombectomy (EVT) for treatment of acute ischemic stroke (AIS),^1^^,^^2^ successful revascularisation is not achieved in 15%–40% of cases,^3^^,^^4^ and at most 60% of patients achieve reperfusion after a single EVT attempt.^4^ This first-pass reperfusion (FPR) is strongly associated with improved functional outcomes and reduced mortality, underscoring the need to better understand determinants of EVT success.

Previous studies have suggested that thrombus composition influences EVT outcomes: red blood cell (RBC)-rich thrombi have been associated with higher rates of FPR,^5^^,^^6^ whereas fibrin- and platelet-rich thrombi have been linked to more challenging retrieval and poorer revascularisation outcomes.^7^ However, these associations have not been consistently observed across studies, and higher RBC content has also been associated with increased risk of intracranial haemorrhage.^8^

These inconsistencies suggest that dichotomous classification of thrombi based on composition does not fully capture their response during mechanical retrieval. Mechanical characteristics, such as thrombus stiffness, may represent a more direct determinant of thrombus–device interaction and EVT efficacy.

Experimental studies using *in vitro* clot analogues have indeed demonstrated that thrombus stiffness affects EVT performance.^9^^,^^10^ However, few studies have characterised *ex vivo* stiffness of AIS thrombi,^11^^,^^12^ and none have systematically examined its relationship with EVT success or related thrombus stiffness with clinical or radiological features.

This prospective observational study examines pre-interventional clinical and radiological markers and histological determinants of thrombus stiffness. It further evaluates the relationship between thrombus stiffness and EVT success, in terms of FPR and procedural complications.

## Patients and methods

### Clinical studies parameters

The prospective single-centre observational study "Mechanical Characterisation of Thrombi Retrieved with Endovascular Thrombectomy for Acute Ischemic Stroke" (MR CLOT) collected thrombus samples and imaging data from patients undergoing EVT between August 2022 and July 2023.

Eligible patients: (1) had undergone EVT for AIS due to an occlusive thrombus in the anterior intracranial vasculature, confirmed on admission CT angiography (CTA); (2) had pretreatment CT scans of adequate quality showing an identifiable clot; (3) had a sufficiently sized thrombus retrieved during EVT in at least one procedural pass for mechanical characterisation and histopathology. Patient enrolment was restricted to cases in which thrombus material could be collected and processed within the predefined experimental workflow and time constraints of the study. Because mechanical characterisation required rapid transfer, preparation and testing of the retrieved thrombus, only patients for whom this experimental workflow could be completed were included. Having received tissue plasminogen activator (tPA) before EVT was not an exclusion criterion. Additional details on inclusion and exclusion criteria are provided in Supplementary Information (SI) S.1.

Treatment success was evaluated by the treating interventionalists based on digital subtraction angiography (DSA) with the expanded Thrombolysis in Cerebral Infarction (eTICI) score,^13^^,^^14^ which measures the reperfusion in the downstream territory of the original occlusion. First-pass reperfusion (FPR) was defined as achieving eTICI scores of 2c (90%–99%, excellent reperfusion) or 3 (100%, complete reperfusion) after the first EVT attempt; a modified FPR (mFPR) was further defined as achieving an eTICI score ≥2b (50%–89%, successful reperfusion) after the first EVT attempt, to include optimal recanalisation in the successful treatment outcomes.^15^

Intravenous thrombolysis (IVT) was administered to all eligible patients according to current guidelines, within 4.5 hours from symptom onset or last known well. Patients who did not receive IVT were outside the therapeutic window or had other contraindications.^16^^,^^17^

Procedural complications were defined as any intraprocedural adverse event recorded during EVT, including embolic events (distal thrombus or embolisation to a new vascular territory), significant flow-limiting vasospasm, arterial dissection or other reported complications.

### Thrombus collection and sample preparation

Thrombus material collection during EVT followed a per-pass protocol, with samples collected separately after each procedural attempt (Figure 1). To preserve sample integrity, the retrieved fragments were stored in containers filled with Dulbecco’s Modified Eagle Medium (DMEM) and maintained at a temperature of 4–10 °C. Following the procedure, the neurointerventionalist completed an intervention form, recording key procedural details, including frontline strategy, number of device passes, the eTICI score and time from arterial puncture to reperfusion. Multiple samples per patient were prepared for mechanical analysis, and all underwent testing within 6 hours from retrieval (SI S.2).

**Figure 1:**
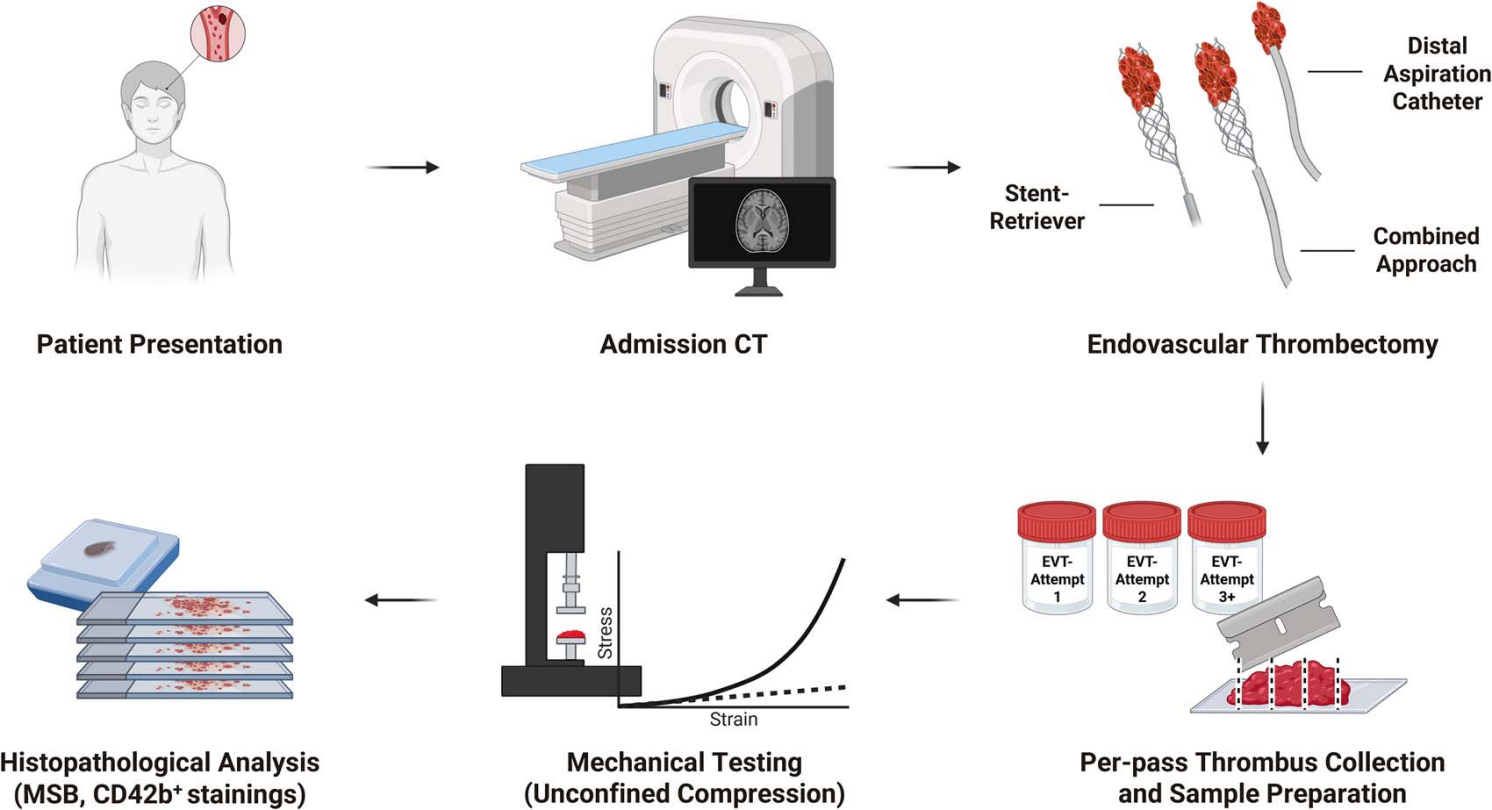
MR CLOT study workflow: Thrombi retrieved from patients undergoing endovascular thrombectomy due to an occlusion in the anterior intracranial vasculature, as confirmed on admission CT angiography, have been collected per attempt and have undergone mechanical (unconfined compression) and histological (MSB, CD42b^+^ stainings) characterisation. Abbreviations: MR CLOT = Mechanical Characterisation of Thrombi Retrieved with Endovascular Thrombectomy for Acute Ischemic Stroke; CT = computed tomography; MSB = Martius-Scarlet-Blue.

### Mechanical characterisation of AIS thrombi

Thrombus material was collected from 73 patients during EVT, with multiple samples obtained per pass for each patient. Adequately sized samples were prepared into 346 test sections (median per patient = 6.5, IQR: 4–11) for mechanical testing,^11^ measuring thrombus stiffness as resistance to deformation under loading.^18^ Briefly, unconfined compression experiments were performed with a custom-built test setup,^11^ with the samples submerged in DMEM at body temperature (37 °C) during the experiment. Force and displacement were recorded and converted into nominal stress–strain values using the sample cross-sectional area. Stiffness at 10% strain was calculated through linear fitting of the initial 5%–15% of the stress–strain curve (SI S.2).

### AIS thrombus histopathological composition

Thrombus histological characterisation was performed following a previously described protocol.^19^^,^^20^ In total, 651 Martius-Scarlet-Blue (MSB)-stained sections were analysed to quantify RBCs and FP aggregates, and 642 CD42b^+^-immunostained sections were analysed to identify platelets. For each mechanical sample, two histological slides were prepared — one for MSB and one for CD42b^+^ staining — each containing two sections. The mean composition of the four sections (two per staining type) was used in statistical analyses. Quantification was expressed as the mean percentage area occupied by each component, thereby accounting for differences in section size (SI S.3).

### CT-based quantitative thrombus imaging characteristics

Pre-interventional non-contrast CT (NCCT) and CTA thin-slice (≤3 mm) images were acquired. The image analysis protocol, including automatic rigid registration, manual marker placement to localise the clot and the ICA terminus, and automatic extraction of CT-based thrombus characteristics, was adopted from a previous work.^21^ The following radiological thrombus characteristics^22^ were measured: thrombus length, thrombus density on NCCT, thrombus perviousness (density difference between CTA and NCCT, a proxy for thrombus permeability),^21^^,^^23^ and the distance (DT) from the ICA terminus to the thrombus (SI S.4).

### Statistical analyses: (generalised) linear mixed-effects regression

Given the hierarchical structure of the dataset (multiple thrombus samples collected per patient for each EVT attempt), analyses were performed using linear mixed-effects regression (LMR) and generalised LMR (GLMR) models. Random intercepts accounted for patient-level clustering,^11^ allowing each patient to have a distinct baseline level for the dependent variable (i.e., thrombus stiffness or EVT success). To satisfy LMR assumptions of normality and homoscedasticity, thrombus stiffness values were log_10_-transformed.

Univariable and multivariable LMR assessed associations between thrombus stiffness and clinical variables (age, sex, occlusion location), radiological features (NCCT density, length, perviousness, DT) and histological composition (RBCs, FP, platelets), with predictors included based on prior clinical knowledge.^11^^,^^24^ Results are reported as β estimates for continuous variables and estimated marginal means for categorical variables, with corresponding 95% CIs. Differences were considered statistically significant when the 95% CI of the contrast did not include zero. Model performance was evaluated using marginal (fixed-effects) and conditional R^2^ (mixed-effects), with bootstrapped decomposition to quantify predictor contributions in partial and importance R^2^, respectively. Multivariable LMR accounted for potential confounding by patient age, sex, occlusion location and thrombus histological and radiological characteristics to identify the main predictors of thrombus stiffness.

The association between thrombus stiffness and EVT success (probability of achieving mFPR) was assessed using univariable and multivariable GLMR (binomial distribution, logit link). This model was adjusted for age, sex, occlusion location, first-pass EVT method and thrombus histological composition (i.e., RBCs, FP and platelets). Occlusion location was included as a confounder,^3^ and histological composition was included to assess whether the association between thrombus stiffness and mFPR persisted independently of thrombus composition. Interaction terms were subsequently added to investigate effect modification, and removed if interactions did not provide significant evidence of effect modification to avoid additional model complexity. The GLMR included random intercepts and slopes for thrombus stiffness by patient, accounting for inter-patient variability in baseline outcome probability and stiffness effect, and clustering of multiple samples per patient. Results are reported as estimated marginal probabilities with corresponding 95% CIs.

All statistical analyses were performed using JASP 0.19.3, except R^2^ metrics, which were computed using RStudio 2024.12.0. Reporting follows the STROBE guidelines.

## Results

### Study population, baseline characteristics and interventional data

A total of 73 AIS patients undergoing EVT were enrolled for screening. Of these, 58 patients with complete radiological, histological and mechanical datasets were available for analysis. Fifteen patients were excluded due to missing thrombus material suitable for characterisation (11%, 8/73) or technical limitations in multimodal data acquisition (9%, 7/73). A detailed overview of patient inclusion and exclusion is provided in the STROBE-compliant flow diagram (SI S.1). Among the remaining 58 patients, 7/73 (9%) had multiple occlusion locations on admission CTA and were excluded from the final analysis as thrombus material could not be assigned to a specific occlusion site. Among the 7 patients with occlusions at multiple locations, 4 had concomitant ICA and M1 occlusions, 1 had ICA and M2 occlusions, 1 had M1 and M2 occlusions, and 1 had M2 and A3 segment of the anterior cerebral artery occlusions. A sensitivity analysis to assess thrombus stiffness variability in patients with double occlusions is reported in SI S.1.

Clinical parameters (age, sex, occlusion location), pre-treatment National Institutes of Health Stroke Scale (NIHSS), treatment methods, procedure duration, EVT attempts, eTICI score post-intervention and after EVT first-pass, and (modified) FPR are summarised in Table 1.

**Table 1:** Baseline characteristics and interventional data: values are numbers (%) of patients, unless stated otherwise. Data refers to 51/73 patients included in the study.

Baseline characteristics and interventional data			
**Median (IQR) age, y**			77 (66–83.5)
**Sex (female)**			34 (66.6%)
**Median (IQR) NIHSS on admission**			16 (11.2–18.7)
**Target occlusion**			
** ICA**			9 (17.6%)
** M1**			25 (49.0%)
** M2**			17 (33.3%)
**Median (IQR) EVT duration, min**			31 (23.5–53.5)
**EVT method**			
** Stent retriever-only**			38 (74.5%)
** Aspiration-only**			0 (0%)
** Combined**			13 (25.5%)
**EVT attempts**	**1** **2** **3** **4** **5** **6**		32 (62.7%) 10 (19.6%) 5 (9.8%) 2 (3.9%) 2 (3.9%) 0 (0%)
**eTICI score**		**Final**	**1^st^ EVT attempt**
	**0** **1** **2A** **2B** **2C** **3**	0 (0%) 0 (0%) 0 (0%) 7 (13.7%) 10 (19.6%) 34 (66.7%)	9 (17.7%) 4 (7.8%) 6 (11.8%) 4 (7.8%) 4 (7.8%) 24 (47.1%)
**First pass reperfusion (FPR)**		**(Unmodified) FPR**	**Modified FPR (mFPR)**
	**0** **1**	23 (45.1%) 28 (54.9%)	19 (37.3%) 32 (62.7%)

Abbreviations: y = years; min = minutes; eTICI = expanded Thrombolysis in Cerebral Infarction; EVT = endovascular thrombectomy; ICA = intracranial carotid artery; IQR = interquartile range; M1, M2 = segments of the middle cerebral artery; NIHSS = National Institutes of Health Stroke Scale.

Corresponding characteristics of the initially enrolled cohort are provided in SI S.1 for comparison. Baseline and procedural characteristics of the included cohort were comparable to those of the initially enrolled population, including similar age, sex distribution and stroke severity.

Within the final cohort, most occlusions occurred in the middle cerebral artery (MCA), including 49% (25/51) in the M1 segment and 34% (17/51) in the M2 segment, while ICA terminal occlusions accounted for 17% (9/51) of patients.

IVT status was available for 40 patients. Among these, 55% (22/40) received IVT, including 50% (20/40) treated at a peripheral hospital prior to transfer and 5% (2/40) treated locally at the stroke centre, while 45% (18/40) did not receive IVT. First-line EVT strategies included stent retriever-only in 74% (38/51) of cases, or a combined approach in 26% (13/51).

In the analytic cohort, 51 patients underwent 84 EVT passes, yielding 139 thrombus fragments and 281 mechanically tested specimens; the corresponding per-patient and per-pass distributions are detailed in SI S.1.

A single EVT attempt was performed in 63% (32/51) of cases, while 37% (19/51) required more attempts. Final eTICI scores indicated excellent reperfusion in 86% (44/51) of patients, including 66% (34/51) achieving complete reperfusion. Overall, 55% (28/51) achieved FPR, while 62% (32/51) achieved modified FPR (mFPR).

For ICA occlusions, stent retriever-only was used as first-line treatment in all patients, with 44% (4/9) achieving FPR. For M1 occlusions, stent retriever-only was used in 56% (14/25) of cases, with 57% (8/14) achieving FPR, while a combined approach in the remaining patients resulted in FPR in 54% (6/11) of cases. For M2 occlusions, stent retriever-only was used in 88% (15/17) of cases, with 53% (8/15) achieving FPR, whereas the combined approach achieved FPR in 100% (2/2) of cases.

Procedural complications occurred in 18% (9/51) of EVT procedures. The most common were embolic complications (10%, 5/51), followed by significant vasospasm (6%, 3/51) and arterial dissection (2%, 1/51).

### Thrombus stiffness by age, sex and occlusion location in AIS patients

A total of 281 thrombus samples retrieved from 51 AIS patients showed a median stiffness of 0.594 kPa (95% CI, 0.208–1.115), showing high inter-patient variability (coefficient of variation = 95%) and intra-patient variability (mean coefficient of variation among patients = 80%), as reported in SI S.2.

Marginal β estimates from the mixed-effects models evaluating the associations between thrombus stiffness and clinical variables (age, sex and occlusion location) are summarised in Table 2. The multivariable LMR explained thrombus stiffness variability with a moderate effect size (marginal R^2^ = 0.236, 95% CI, 0.089–0.545), increasing when accounting for patient-level clustering (conditional R^2^ = 0.567), reflecting the high explanatory power of the mixed-effects model.

**Table 2:** Univariable and multivariable^a^ linear mixed regression (LMR) assessed associations between clinical, radiological and histological thrombus characteristics and thrombus stiffness in 281 thrombi from 51 patients.

	Model term		Unadjusted estimate (95% CI)	Adjusted ^a^ estimate (95% CI)	Partial / marginal R ^2^ (95% CI)	Importance /conditional R ^2^ (95% CI)
**Clinical** **variables**	**Age (y)**		0.001 (−0.009 to 0.011)	−0.026 (−0.582 to 0.585)	0 (0–0.389)	0 (0–0.147)
	**Sex**	**Female**	−0.160 (−0.337 to 0.017)	−0.170 (−0.379 to 0.040)	0.008 (0–0.395)	0.032 (0–0.210)
		**Male**	−0.415 (−0.661 to −0.168)	−0.343 (−0.611 to 0.076)		
	**Occlusion location**	**ICA**	0.044 (−0.243 to 0.332)	−0.041 (−0.440 to 0.359)	0.025 (0–0.406)	0.147 (0.002–0.348)
		**M1**	−0.119 (−0.303 to 0.065)	−0.118 (−0.383 to 0.147)		
		**M2**	−0.627 (−0.860 to −0.393)	−0.610 (−0.992 to −0.228)		
**Clinical predictors**					**0.070** (0–0.436)	**0.179**
**Radiological variables**	**NCCT density (HU)**		0.010 (−0.004 to 0.025)	0.010 (−0.011 to 0.031)	0.004 (0–0.392)	0.053 (0–0.147)
	**Length (mm)**		0.003 (−0.011 to 0.017)	−0.007 (−0.022 to 0.008)	0.004 (0–0.392)	0 (0–0.124)
	**Perviousness (HU)**		−0.007 (−0.016 to 0.002)	−0.001 (−0.010 to 0.008)	0.005 (0–0.393)	0.039 (0–0.247)
	**DT (mm)**		−0.009 (−0.016 to −0.002)	−0.002 (−0.009 to 0.013)	0 (0–0.387)	0.088 (0–0.280)
**Radiological predictors**					**0.027** (0–0.408)	**0.180**
**Clinical and radiological predictors**					**0.166** (0.019–0.499)	**0.359**
**Histological variables**	**RBCs (%)**		−0.005 (−0.008 to −0.001)	−0.002 (−0.009 to 0.004)	0.004 (0–0.392)	0.055 (0–0.230)
	**FP (%)**		0.003 (−0.001 to 0.006)	0.001 (−0.004 to 0.007)	0.004 (0–0.392)	0.045 (0–0.197)
	**Platelets (%)**		0.006 (0.002–0.010)	0.004 (−0.001 to 0.010)	0.008 (0–0.395)	0.078 (0–0.242)
**Histological determinants**					**0.065** (0–0.432)	**0.178**
**Clinical, radiological predictors and histological determinants**					**0.236** (0.089–0.545)	**0.567**

a Adjusted for clinical, radiological and histological variables. A random intercept for patient accounts for multiple thrombi per individual. Estimated β trends and marginal means estimates are reported for continuous and categorical variables, respectively. Abbreviations: y = years; HU = Hounsfield unit; mm = millimeter; DT = distance from ICA terminus to thrombus proximal end; FP = fibrin/platelets; ICA = intracranial carotid artery; M1, M2 = middle cerebral artery; NCCT = non-contrast CT; RBCs = red blood cells. Bold values indicate the R^2^ values for the overall predictor categories.

No statistically significant differences in thrombus stiffness have been found across age groups. Regarding sex, thrombus stiffness was higher in women (0.692 kPa, 95% CI, 0.460–1.040) compared to men (0.384 kPa, 95% CI, 0.218–0.679), with a difference of 0.308 kPa (95% CI, -0.219–0.822).

Regarding occlusion location (Figure 2), thrombi retrieved from the proximal M2 segment of the middle cerebral artery showed significantly lower stiffness (0.236 kPa, 95% CI, 0.136–0.409) compared to those from the M1 segment (0.760 kPa, 95% CI, 0.492–1.174) and the ICA (1.106 kPa, 95% CI, 0.558–2.192). The difference in stiffness between M1 and proximal M2 was 0.524 kPa (95% CI, 0.093–1.024), corresponding to a statistically significant 3.22-fold increase (95% CI, 1.23–8.42). For ICA versus proximal M2, the difference was 0.870 kPa (95% CI, 0.166–2.009), indicating a significant 4.68-fold increase (95% CI, 1.41–15.55), while ICA vs M1 showed a non-significant difference of 0.346 kPa (95% CI, -0.591 to 1.649), corresponding to a 1.45-fold increase (95% CI, 0.49–4.31).

**Figure 2:**
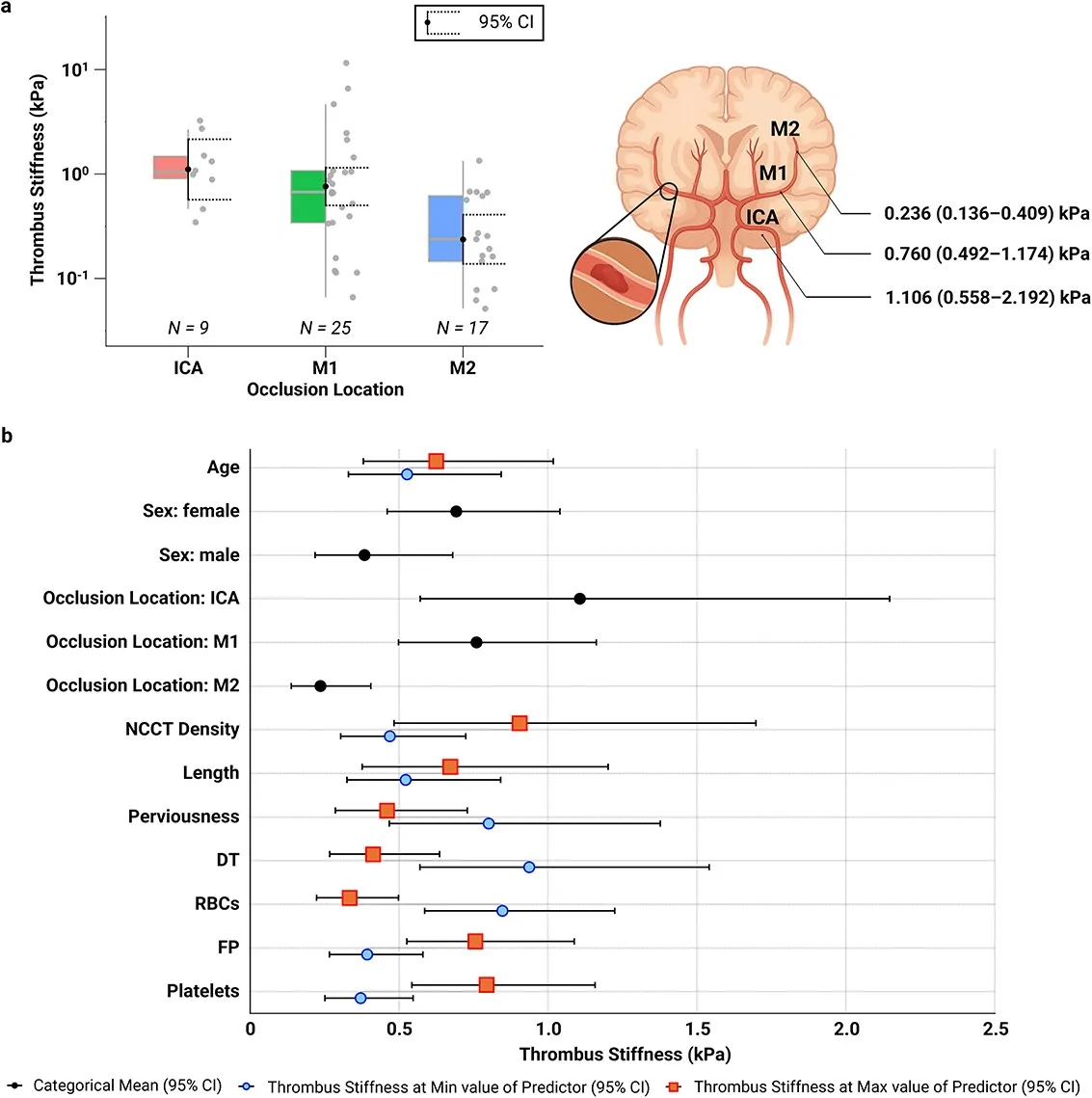
Thrombus stiffness across occlusion location and clinical, radiological and histological predictors: a) univariable linear mixed-effects regression of thrombus stiffness by occlusion location in 281 thrombus samples from 51 patients. Left: Log₁₀-transformed stiffness; right: Absolute values (kPa). Grey lines indicate fixed-effect predictions; black lines indicate marginal mean predictions accounting for multiple samples per patient. b) Estimated marginal means of thrombus stiffness across clinical, radiological and histological predictors from the same linear mixed-effects regression analyses, shown with 95% CI. Continuous variables are displayed at minimum (blue circles) and maximum (orange squares) values; categorical variables are shown by category mean estimates. Abbreviations: ICA = intracranial carotid artery; M1, M2 = middle cerebral artery; NCCT = non-contrast computed tomography; DT = distance from ICA terminus to thrombus proximal end. RBCs = red blood cells; FP = fibrin/platelets aggregates.

No differences in thrombus composition across occlusion locations were observed (SI S.3), confirming occlusion-site differences in thrombus stiffness regardless of thrombus histopathological composition.

### Association between thrombus stiffness and pre-interventional imaging and histological characteristics

Associations between thrombus stiffness and radiological characteristics (NCCT density, thrombus length, perviousness and DT) as well as histological composition are examined to identify quantitative imaging markers of thrombus stiffness and to evaluate the influence of thrombus components on its stiffness.

Thrombi from 51 patients showed NCCT density of 45.4 ± 12.3 HU, length of 21.1 ± 12.3 mm, perviousness of 14.3 ± 20.0 HU, DT of 28.4 ± 25.0 mm, with an average thrombus composition of 28.2 ± 21.6% RBCs, 70.2 ± 21.5% FP, and 52.0 ± 17.3% platelets.

Radiological and histological associations with thrombus stiffness are summarised in Table 2, with univariable estimates depicted in Figure 2b.

NCCT density showed a non-significant positive relationship with thrombus stiffness (β = 0.010, 95% CI, -0.004 to 0.025), with stiffness ranging from 0.469 kPa (95% CI, 0.304–0.724) at the minimum NCCT density of 40.8 HU to 0.905 kPa (95% CI, 0.483–1.698) at the maximum of 65.3 HU. Thrombus length was not associated with stiffness (β = 0.003, 95% CI, -0.011 to 0.017). Thrombus perviousness exhibited a non-significant negative relationship (β = -0.007, 95% CI, -0.016 to 0.002), with stiffness ranging from 0.801 kPa (95% CI, 0.467–1.377) at the lowest perviousness value to 0.457 kPa (95% CI, 0.286–0.729) at the highest value of 25.7 HU. DT was significantly negatively associated with stiffness, ranging from 0.937 kPa (95% CI, 0.580–1.541) at minimum DT of 3.96 mm to 0.413 kPa (95% CI, 0.267–0.636) at maximum DT of 42.71 mm. Among imaging variables, DT showed the largest relative variation in stiffness, corresponding to a 2.3-fold decrease across its range.

RBC content demonstrated a negative association (β = -0.005, 95% CI, -0.008 to -0.001), with stiffness ranging from 0.847 kPa (95% CI, 0.586–1.224) at minimum RBC content of 12.6% to 0.334 kPa (95% CI, 0.223–0.498) at maximum RBC content of 61.5%. FP showed a positive, non-significant trend, with stiffness varying from 0.393 kPa (95% CI, 0.266–0.580) at minimum FP content of 37.7% to 0.756 kPa (95% CI, 0.526–1.088) at maximum FP content of 85.4%. Platelet content was significantly positively associated with thrombus stiffness (β = 0.006, 95% CI, 0.002–0.010), with stiffness ranging from 0.371 kPa (95% CI, 0.251–0.547) at minimum platelet content of 29.1% to 0.794 kPa (95% CI, 0.543–1.158) at maximum platelet content of 72.2%.

### Clinical, radiological and histological predictors of thrombus stiffness

The variance of log₁₀-transformed thrombus stiffness was 0.394: the full model, including all predictors, was able to estimate 56.7% of this variance (2.97-fold spread in thrombus stiffness), with similar contributions from clinical, radiological and histological variables (17.9%, 18.0%, 17.8%, respectively). Marginal and conditional R^2^ values are summarised in Table 2.

Among all the predictors, occlusion location explained a substantial proportion of this variance, with thrombi from the proximal M2 segment showing the highest importance R^2^, accounting for approximately 14.7% (95% CI, 0.002–34.8) of the variability in stiffness.

Among the radiological predictors, DT explained 8.8% of thrombus stiffness variance (95% CI, 0–28), confirming the importance of occlusion site as clinical and radiological marker. Thrombus NCCT density (5.3%, 95% CI, 0–14.7) and perviousness (3.9%, 95% CI, 0–24.7) followed, while thrombus length contributed minimally.

Among the histological thrombus components, platelets exhibited the highest explanatory power for thrombus stiffness, accounting for 7.8% of the variance (95% CI, 0–24.2), followed by RBCs (5.5%, 95% CI, 0–23) and FP (4.5%, 95% CI, 0–19.7).

Occlusion location, specifically M2, and more precisely, DT were the strongest predictors of thrombus stiffness variability, independent of thrombus histopathological composition or radiological characteristics.

### Clinical relevance of thrombus stiffness in EVT success

Adjusted marginal estimates from the multivariable GLMR assessing the association between thrombus stiffness and the probability of achieving a successful EVT outcome, defined as mFPR, are summarised in Table 3.

**Table 3:** Association between thrombus stiffness and intervention success: associations were examined using a multivariable GLMR including 281 thrombus samples from 51 patients. Marginal estimated probabilities of positive mFPR with 95% CI are shown.

Association thrombus stiffness and intervention technical outcome			
	Model term		Marginal ^a^ estimated probability of positive mFPR (95% CI)
**Clinical variables**		Lower bound = 54 y	0.580 (0.357–0.774)
	**Age**	Median = 71 y	0.708 (0.517–0.885)
		Upper bound = 88 y	0.809 (0.608–0.921)
		Male	0.835 (0.631–0.938)
		Female	0.535 (0.341–0.720)
		ICA	0.448 (0.245–0.670)
	**Occlusion location**	M1	0.917 (0.795–0.970)
		M2	0.611 (0.308–0.847)
**Procedural variables**		SR only	0.750 (0.626–0.843)
		Combined (SR + aspiration)	0.787 (0.464–0.940)
**Mechanical variables**		Lower bound = 0.165 kPa	0.779 (0.484–0.929)
	**Thrombus stiffness**	Median = 0.696 kPa	0.708 (0.517–0.846)
		Upper bound = 2.92 kPa	0.625 (0.343–0.842)
**Histological variables**		Lower bound = 11.8%	0.657 (0.427–0.831)
	**RBCs**	Median = 36.0%	0.708 (0.517–0.846)
		Upper bound = 60.2%	0.753 (0.522–0.895)
		Lower bound = 38.9%	0.742 (0.480–0.899)
	**FP**	Median = 62.6%	0.707 (0.516–0.846)
		Upper bound = 86.2%	0.671 (0.408–0.858)
		Lower bound = 29.6%	0.752 (0.525–0.892)
	**Platelets**	Median = 51.7%	0.708 (0.517–0.846)
		Upper bound = 73.8%	0.659 (0.428–0.834)

a Adjusted for age, sex, occlusion location, first-line endovascular thrombectomy method and thrombus histological composition. Estimated marginal means adjust for all covariates except the variable of interest. The model includes random intercepts and slopes for log₁₀-transformed thrombus stiffness at the patient level. Abbreviations: y = years; CI = confidence interval; GLMR = generalised linear mixed regression; mFPR = modified first-pass reperfusion (eTICI ≥ 2b).

Direct association between thrombus stiffness and mFPR was not significant, negative unadjusted (β = -0.941, 95% CI, -6.669 to 4.787) and adjusted (β = -0.640, 95% CI, -2.232 to 0.952) estimates.

Female AIS patients had a lower likelihood (adjusted probability) of achieving mFPR (53.5%, 95% CI, 34.1–72.0) than male patients (83.5%, 95% CI, 63.1–93.8), independent of age, occlusion location, first-pass EVT method, thrombus composition and stiffness, although the interaction between thrombus stiffness and patient sex was not statistically significant.

No differences in EVT success were observed regarding first-line EVT methods, with the combined approach leading to slightly higher mFPR probability with respect to stent retriever only interventions (0.787, 95% CI, 0.464–0.940 vs 0.750, 95% CI, 0.626–0.843).

Given the known influence of occlusion location on EVT success,^13^ a further stratification was adopted using marginal estimates by occlusion site (Figure 3): mean adjusted probability of mFPR was 40.4% (95% CI, 24.5–67.0) for thrombi retrieved from the ICA, 89.7% (95% CI, 79.5–97.0) for M1 occlusions, and 53.0% (95% CI, 30.8–84.7) for proximal M2 occlusions, across the corresponding stiffness ranges. The interaction between thrombus stiffness and occlusion location on EVT success was not statistically significant. Overall, mFPR probability was lower in the stiffest thrombi from the ICA and in the softest from the proximal M2 segment compared with those from M1 occlusions.

**Figure 3:**
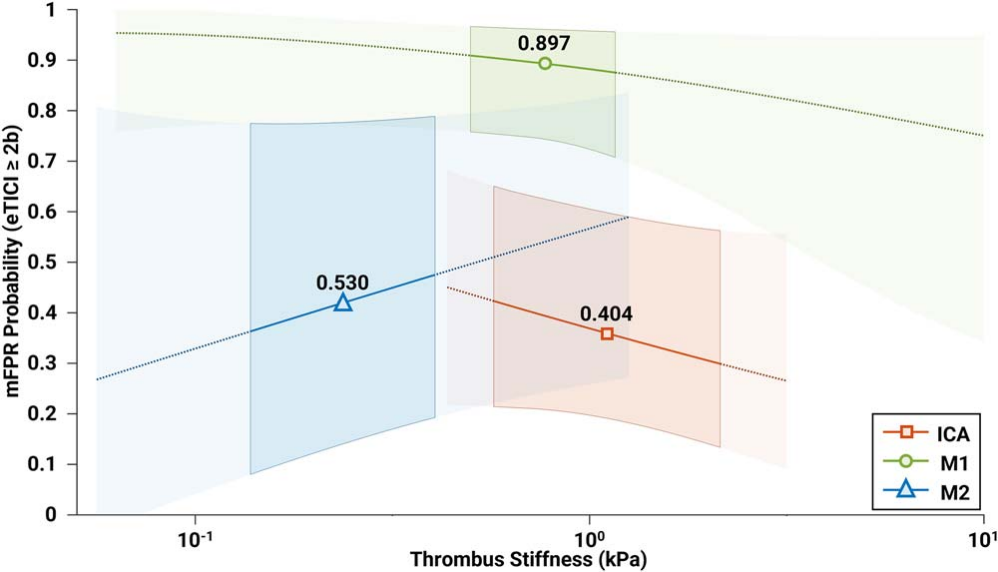
Thrombus stiffness influence on endovascular thrombectomy (EVT) success among occlusion locations: results from multivariable generalised linear mixed-effects regression, adjusted for age, sex, first-pass EVT method and thrombus histological composition, showing a decrease in the probability of mFPR for softer thrombi in the proximal M2 segment and for high-stiffness thrombi in the ICA. Bold regression lines and solid 95% CI represent the stiffness range derived from the full LMR model that accounts for clustering of multiple thrombi per patient. Regression lines with semi-transparent confidence bands correspond to the stiffness range obtained by averaging thrombus stiffness per patient. Abbreviations: ICA = intracranial carotid artery; M1, M2 = middle cerebral artery; mFPR = modified first-pass reperfusion (eTICI ≥ 2b).

The GLMR between thrombus stiffness and FPR showed results similar to those for mFPR (SI S.5). Expanding the success criterion from FPR (eTICI≥2c) to mFPR (eTICI≥2b) provided better stratification of procedural success across occlusion sites. Specifically, while M1 occlusions showed similar first-pass success probabilities under both definitions, ICA and M2 occlusions exhibited clearly lower success rates when considering mFPR.

In addition to EVT success, other procedural indicators were assessed with respect to thrombus stiffness. Thrombi retrieved from patients experiencing procedural complications were significantly softer than those from patients without complications. No association was found between thrombus stiffness and IVT administration, number of EVT attempts or intervention duration (SI S.5).

## Discussion

### Can occlusion site serve as indicator of thrombus stiffness in EVT?

Multivariable LMR, incorporating clinical, radiological and histological thrombus characteristics, highlighted the predictive value of readily available clinical data, such as occlusion location and patient sex, in estimating thrombus stiffness. The addition of radiological variables further improved model performance, underscoring the incremental value of imaging biomarkers alongside clinical predictors. Notably, the explanatory power of clinical variables alone was comparable to that of *ex vivo* histopathological composition.

Importantly, thrombi located in smaller, more distal vessels were significantly softer than those in more proximal arteries, independent of age, sex and thrombus radiological or histological characteristics: stiffer thrombi occluded proximal and larger intracranial arteries, whereas softer thrombi were found in more distal vessels, potentially reflecting a greater propensity for fragmentation and distal embolisation.

Among all evaluated features, occlusion location emerged as the strongest single marker of thrombus stiffness variance, identifying occlusion site as a clinically accessible indicator of thrombus mechanical properties. These findings suggest that the anatomical site of occlusion may provide a readily available indication of the expected mechanical properties of the target thrombus, potentially relevant for individualised thrombectomy strategies. However, external validation in an independent AIS cohort would be necessary to assess the generalizability and predictive value of this association, as the substantial residual variability in stiffness indicates that occlusion site alone does not fully capture the mechanical properties of individual thrombi. Although the full LMR model showed a moderate explanatory power (conditional R^2^ = 0.567), the remaining unexplained variability in thrombus stiffness may reflect AIS aetiology, patient comorbidities or other factors not captured in the present analysis.

While occlusion site remains a primary determinant of thrombus mechanical properties, thrombus stiffness was also higher in female patients compared with male patients, consistent with *in vitro* studies in a healthy population that reported sex-dependent differences,^18^ leading to lower probability of achieving mFPR. Together, these findings suggest that occlusion site and patient sex may influence thrombus mechanics, with potential implications for individualised EVT strategy planning in AIS and design of future devices.

### On the heterogeneous nature of thrombi

Thrombi retrieved from AIS patients exhibited substantial heterogeneity in mechanical properties, histological composition and radiological characteristics, both within individual patients (intra-patient) and across different patients (inter-patient).^7^^,^^25^ Such heterogeneity may influence treatment outcomes through mechanisms related to thrombolysis efficacy and device–thrombus interaction during EVT.^9^^,^^26^ To account for this hierarchical variability, mixed-effects models were employed, incorporating the clustering of multiple samples per thrombus and per patient. This approach significantly improved the explanation of thrombus stiffness variance, with the conditional R^2^ of the full model rising to 56.7%, highlighting the importance of accounting for nested data structures in the biomechanical characterisation of intracranial thrombi. This could also hold true for other soft biological tissues.

### On the role of platelets in thrombus stiffness in AIS

The limited explanatory value of histological variables for thrombus stiffness likely reflects the biological complexity of thrombus composition. Among the components, platelets play a uniquely active role in thrombus mechanics, as supported by preliminary *ex vivo*^5^^,^^9^^,^^11^ and *in vitro* studies.^18^^,^^27^ By contracting and remodelling the fibrin network, platelets compact the thrombus and increase its mechanical stiffness. In contrast, RBCs mainly influence thrombus volume as passive fillers, while fibrin serves as a structural scaffold whose mechanical contribution depends on platelet-driven reorganisation and cross-linking. As such, platelet content emerges as the most influential histological determinant of thrombus stiffness, due to its direct biomechanical impact on thrombus architecture. Nevertheless, the interplay among thrombus components may reflect the microstructural heterogeneity driving differences in mechanical properties. This may explain the lack of association in this cohort between thrombus stiffness and pre-interventional imaging markers. Radiological metrics, such as NCCT thrombus density and perviousness, primarily capture bulk thrombus composition, particularly RBC-driven attenuation. In contrast, platelet-rich thrombi are typically isodense relative to surrounding brain parenchyma on NCCT,^28^ reflecting microstructural platelet-mediated fibrin remodelling that governs thrombus mechanical response.^29^

Different histological determinants may underlie other thrombus mechanical properties. For example, thrombus toughness, reflecting resistance to rupture, has been linked to RBCs content.^18^ Notably, the GLMR relating thrombus stiffness to EVT success showed comparable contributions from both RBCs and platelets, suggesting that multiple components may jointly influence thrombus biomechanical properties and clinical response to treatment.

### Clinical impact of thrombus stiffness variation on EVT treatment

In this cohort, first-pass EVT showed lower success rates in ICA and M2 occlusions. Thrombi from the ICA were significantly stiffer compared to thrombi from M2. These observations suggest that the most difficult thrombi to retrieve in the anterior circulation are the stiffest ones in proximal occlusions, and the softest thrombi in distal occlusions, indicating that different EVT strategies tailored to thrombus stiffness differences across occlusion sites may be appropriate for different vascular territories in the anterior circulation.^30^^,^^31^

Furthermore, softer thrombi were associated with procedural complications, most commonly embolic events, supporting the hypothesis that reduced stiffness may increase susceptibility to thrombus fragmentation and distal embolisation during EVT.^32^^,^^33^

### Study limitations and considerations

Since *ex vivo* thrombi can only be obtained from successful EVT procedures, the analysis excludes those most resistant to thrombectomy, where thrombus retrieval was unsuccessful. These may represent either the stiffest or the softest and most embolic thrombi, which are the most challenging to retrieve and therefore underrepresented in analyses. This might have introduced selection effects with respect to patients with unsuccessful thrombus retrieval, as well as those in whom insufficient thrombus material or complete multimodal data were unavailable. For the analysis of occlusion location and thrombus stiffness, these exclusions were not based on occlusion location and therefore do not directly involve selection on the exposure of interest. In contrast, selection may be more consequential for the analysis relating thrombus properties to procedural success, as thrombus availability may be related to procedural outcome. Although comparisons between the initially enrolled and final cohorts did not indicate major differences in the available demographic and baseline clinical characteristics, differences were observed in several procedural and reperfusion characteristics. Selection based on unmeasured thrombus-specific properties cannot be excluded. The findings should therefore be interpreted in the context of this potential bias.

Although fibrinolysis has been shown to reduce thrombus stiffness and mechanical integrity in experimental studies,^34^^,^^35^ potential lysis-related effects may have remained indistinct in this cohort, as most IVT was administered at peripheral hospitals prior to transfer and the delay between thrombolysis and thrombus mechanical characterisation after EVT may have attenuated detectable lysis-related changes in thrombus stiffness.

Given that EVT-retrieved thrombi with characterised mechanical properties are scarce, and their structural and compositional heterogeneity can substantially affect their mechanical response, this study prioritised testing multiple samples from the same patients rather than expanding the number of patients. Multiple specimens per patient were used to characterise within-patient heterogeneity in thrombus mechanical properties, but were not considered independent mFPR outcomes. Accordingly, the effective number of independent observations for the outcome analysis remained 51 patients. Consequently, while the LMR analysis showed a moderate effect size supporting the robustness of the results, the GLMR analysis produced a small effect size and was based on a relatively limited number of independent outcome observations. Therefore, the GLMR results should be interpreted as exploratory and hypothesis-generating.

## Conclusion

Thrombi in distal intracranial vessels up to proximal M2 are significantly softer than those in proximal arteries of the anterior cerebral circulation. First-pass EVT success is lower for the stiffest thrombi in the ICA and for the softest thrombi in M2, underscoring the need to optimise thrombectomy techniques and devices based on the mechanical properties of the thrombus.

## Supplementary Material

Supplementary_material_aakag132

## Data Availability

The datasets generated during and/or analysed during the current study are available from the corresponding author on reasonable request.

## Acronyms and abbreviations

AIS, acute ischemic stroke; CI, confidence interval; CT, computed tomography; CTA, computed tomography angiography; DT, distance from intracranial carotid artery terminus; eTICI, expanded thrombolysis in cerebral infarction; EVT, endovascular thrombectomy; FP, fibrin/platelets aggregates; FPR, first-pass reperfusion; GLMR, generalised linear mixed-effects regression; ICA, intracranial carotid artery; IVT, intravenous thrombolysis; LMR, linear mixed-effects regression; mFPR, modified first-pass reperfusion; NCCT, non-contrast computed tomography; RBCs, red blood cells.
