# Supplementary material for: Thrombus stiffness depends on occlusion site and influences thrombectomy outcomes in acute ischemic stroke
Source: Eur Stroke J. 2026 Nov 9;11(11):aakag132. doi: 10.1093/esj/aakag132 (PMC13747018; doi:10.1093/esj/aakag132)
Supplement: Supplementary file 1 [file supplementary_material_aakag132.zip › ESJ_Supplementary_revised_aakag132.docx]

**Supplementary Information**

**Thrombus stiffness depends on occlusion site and influences thrombectomy outcomes in acute ischemic stroke**

Short title:

**Occlusion site-dependent thrombus stiffness in AIS**

**S.1 Study Inclusion, Baseline Characteristics of Patients and Interventional Data**

The STROBE flow diagram in *Fig. S.1* summarizes the patient selection process and reasons for exclusion from the final analysis.

*Fig. S.1 –* ***STROBE flow diagram of patient selection in the MR CLOT study:*** *of 73 AIS patients undergoing EVT between August 2022 and July 2023, 58 had complete multimodal datasets available. Fifteen patients were excluded due to technical limitations affecting radiological, histological, or mechanical data acquisition. Among the remaining patients, 7 with multiple occlusion locations on admission CT angiography were excluded, resulting in a final cohort of 51 patients included in the analysis. The diagram summarizes the study inclusion criteria, exclusion steps, and derivation of the final study population.*

Baseline characteristics of the 73 patients enrolled in the study are summarized in *Table S.1* for comparison with the inclusion cohort (*Table 1*).

Baseline and procedural characteristics of the included cohort were comparable to previously reported AIS cohorts, supporting the representativeness of this study population. The median age of this cohort was higher than in most EVT randomized trials^1^ but better reflects the older age distribution of real-world AIS populations.^2^

To assess whether the exclusion of 22 of the 73 enrolled patients (30%) resulted in systematic differences between the analysed cohort and the enrolled population, baseline and procedural characteristics were formally compared between the 51 included and 22 excluded patients. No significant differences were observed in age (median [IQR], 77 [66.0–83.5] vs. 80 [73.0–85.8] years; Mann–Whitney U test, *p*=0.548), sex (67% vs. 64% female; Fisher exact test, *p*=0.795), or NIHSS on admission (16 [11.2–18.8] vs. 17 [11.2–20.2]; Mann–Whitney U, *p*=0.553). The distribution of EVT methods (stent retriever-only/combined/aspiration-only: 38/13/0 vs. 15/1/1; chi-square test, *p*=0.058) was also not significantly different. Excluded patients, however, had a significantly longer EVT duration (86 [57.0–98.5] vs. 31 [23.5–53.5] min; Mann–Whitney U, *p*=0.007) and required more EVT attempts (2 [1.0–4.0] vs. 1 [1.0–2.0]; Mann–Whitney U, *p*=0.016). They also had lower rates of first-pass reperfusion (FPR: 23% vs. 55%; Fisher exact test, *p*=0.020) and modified first-pass reperfusion (mFPR: 36% vs. 63%; Fisher exact test, *p*=0.045). Thus, the included and excluded groups showed no statistically significant differences and were numerically similar with respect to the assessed demographic and baseline characteristics, while they differed in procedural and reperfusion characteristics. These differences may limit the generalisability of the analysed cohort to the broader enrolled EVT population, but do not by themselves establish selection bias or imply compromised internal validity of the exposure–outcome associations examined within the included cohort.

Angiographic outcomes were assessed by the treating interventionalists using standardized eTICI criteria on procedural digital subtraction angiography. Although independent adjudication was not available, readers were blinded to thrombus stiffness measurements reducing the likelihood of observer bias with respect to the investigated association between thrombus stiffness and EVT success.

Because occlusions at multiple arterial locations could potentially increase thrombus heterogeneity, thrombus stiffness between the seven double-occlusion patients and all single-occlusion patients was compared. Central tendency did not differ between the multisite and the single occlusion location groups (mean stiffness 0.92 kPa vs. 0.73 kPa; Welch's t-test, *p*=0.19). Pooled variance was numerically lower in the multisite group (0.231 vs. 0.424; Levene's test, *p*=0.002), However, within-patient variability, i.e., heterogeneity among fragments from the same patient, was nearly identical between groups (0.171 vs. 0.195; Mann-Whitney U on per-patient coefficient of variation, *p*=0.64). This sensitivity analysis found no evidence that multisite occlusions increase thrombus stiffness variability.

***Table S.1*** – Baseline Characteristics and Interventional Data: values are numbers (%) of patients, unless stated otherwise. Data refers to all 73 patients enrolled in the study; when missing, the number with available data is indicated.

| Baseline Characteristics and Interventional Data | | | |
| --- | --- | --- | --- |
| Median (IQR) age, y | 77 (66–85) | | |
| Sex (female) | 49 (67%) | | |
| Median (IQR) NIHSS on admission | 16 (11–19) | | |
| Target occlusion (N = 66) | | | |
| ICA | 9 (12%) | | |
| M1 | 32 (44%) | | |
| M2 | 25 (34%) | | |
| Median (IQR) EVT duration, min | 40.5 (24.3–66.5) | | |
| EVT Method (N = 68) |  | | |
| Stent retriever-only | 52 (70%) | | |
| Aspiration-only | 2 (3%) | | |
| Combined | 14 (19%) | | |
| EVT Attempts | **1 2 3 4 5 6** | 42 (57%) 11 (15%) 9 (12%) 4 (5%) 6 (8%) 1 (1%) | |
| eTICI score |  | **Final** | **1^st^ EVT attempt** |
|  | **0 1 2A 2B 2C 3** | 2 (3%) 4 (5%) 0 (0%) 14 (19%) 13 (18%) 40 (54%) | 21 (30%) 3 (4%) 5 (7%) 9 (12%) 7 (9%) 28 (38%) |
| First Pass Reperfusion (FPR) |  | **(Unmodified) FPR** | **Modified FPR (mFPR)** |
|  | **0 1** | 38 (53%) 35 (47%) | 29 (40%) 44 (60%) |

IQR: interquartile range; NIHSS: National Institutes of Health Stroke Scale; ICA: intracranial carotid artery; M1, M2: segments of the middle cerebral artery; EVT: endovascular thrombectomy; eTICI: expanded Thrombolysis in Cerebral Infarction.

The hierarchical structure of thrombus acquisition and specimen characterization is summarized in *Fig. S.2*. Overall, 73 patients were enrolled and underwent 143 EVT passes, of which 79 (55%) yielded thrombus material. A total of 183 thrombus fragments were retrieved, corresponding to a median of 2 fragments per pass (IQR 1–3) and 2 fragments per patient (IQR 1–3). From these fragments, 346 specimens were mechanically tested, with a median of 2 specimens per fragment (IQR 1–3) and 6 specimens per patient (IQR 4–11).

After application of the prespecified exclusion criteria, the analytic cohort comprised 51 patients and 84 EVT passes, of which 59 (70%) yielded thrombus material. These passes yielded 139 thrombus fragments, from which 281 specimens underwent mechanical characterization. The median number of EVT passes per patient was 1 (IQR 1–1), and the median number of thrombus fragments per patient was 2 (IQR 1–3). The 281 mechanically characterized specimens corresponded to a median of 2 specimens per fragment (IQR 1–3) and 5 specimens per patient (IQR 3–8). Thus, multiple specimens from an individual patient could originate from the same EVT pass and thrombus fragment, reflecting the hierarchical structure of the dataset.

*Fig. S.2 –* ***Hierarchical structure of thrombus specimen acquisition.*** *Schematic representation of the study cohort and hierarchical progression from patients to EVT passes, thrombus fragments, and mechanically tested specimens in the enrolled and analytic cohorts. Per-patient and per-level distributions are reported as median (IQR) and range. EVT: endovascular thrombectomy; IQR: interquartile range; NIHSS: National Institutes of Health Stroke Scale.*

**S.2 Mechanical Characterization of Acute Ischemic Stroke Thrombi**

After retrieval, thrombus fragments were sectioned into 1–2 mm thick slices to achieve an approximate height-to-diameter aspect ratio of 1.5, minimizing slippage, uneven force distribution, buckling, and barrelling during compression testing. Each specimen was trimmed to obtain two flat, parallel surfaces suitable for testing. Because reliable unconfined compression testing requires flat contact surfaces, mechanical samples were obtained from the central portions of thrombus fragments, while irregular proximal and distal ends were excluded from mechanical testing.

The preparation and testing workflow was time-sensitive, as all mechanical samples had to be processed and tested within 6 hours after thrombus retrieval to ensure mechanical integrity of the samples. Preparation involved manual sectioning and trimming of each sample, followed by mechanical testing. Each compression test lasted approximately 8 minutes, and multiple samples were tested per patient. Given the large number of samples generated during the study (346 test sections from 73 patients), completing this workflow for every patient undergoing EVT was not feasible. Consequently, enrolment was non-consecutive and restricted to cases in which the required thrombus material and experimental processing capacity were available within the predefined time window.

Cross-sectional area (CSA) of each sample was determined from calibrated photographs using ImageJ. After mechanical testing, samples were preserved in HEPES buffer solution (Sigma-Aldrich) and processed for compositional analysis. Proximal and distal fragment ends, together with unused slices, were combined into a single histological specimen to account for potential longitudinal heterogeneity. The preparation workflow is illustrated in *Fig. S.3*.

Fig. S.3 – **Thrombus samples preparation**: a) containers with thrombus fragments per pass; b) mold and blades used for slicing; c) diagram showing thrombus fragment sectioning for mechanical testing; d) Example of thrombus fragments before sectioning; e) Example of thrombus sample for mechanical testing.

To quantify thrombus mechanics, stiffness at 10% strain was evaluated. Mechanical properties were assessed using unconfined compression testing with a previously described custom-built compression device (*Fig. S.4*).^3^ The system incorporated a 2.5 N load cell (LSB200 Jr Miniature S-beam, Futek). Samples were compressed to 80% of their original height over 20 cycles at a strain rate of 0.1 mm/s, while submerged in HEPES buffer at 37°C to approximate physiological conditions.

*Fig. S.4 –* ***Thrombus mechanical testing****:* a) design of the compression tester, with a close-up view of a thrombus sample under the compression plate in the water bath (updated from a previous work^3^); b) force-time signal curve along the twenty compression cycles; c) hysteresis area for the first compression cycle and detail of the first loading curve, showing an initial linear phase, followed by a non-linear stiffening phase, concluding with a higher linear phase.

Force and displacement data were converted to nominal stress–strain values using the measured cross-sectional area. Stiffness at 10% strain was calculated by linear fitting of the 5–15% region of the stress–strain curve, corresponding to the initial elastic deformation phase (*Fig. S.5*).^3^

The mechanical interaction between thrombectomy devices and thrombi during retrieval, particularly with stent retrievers, may alter thrombus structure through compression, stretching, or fragmentation. Therefore, the mechanical properties measured *ex vivo* may not fully reflect the native *in vivo* thrombus stiffness prior to device engagement. Although no differences in thrombus stiffness were observed between thrombi retrieved with stent retriever–only techniques and those retrieved using a combined stent retriever–aspiration approach, the potential influence of device–thrombus interaction on the measured mechanical properties cannot be completely excluded.

*Fig. S.5 –* ***Thrombus Stiffness Variability****:* results from descriptive statistics on 281 samples from 51 patients, showing that thrombi retrieved from AIS patients have high significant inter- and intra-patient variability in stiffness.

**S.3 Histopathological Characterization of Acute Ischemic Stroke Thrombi**

After mechanical testing, thrombus fragments were prepared for histological analysis. Samples were fixed in 3.8–4.2% formaldehyde for 24–48 hours, preventing autolysis and preserving tissue morphology. After fixation, samples were transferred to HEPES buffer and stored at 4–8 °C for a maximum of two weeks before automated processing using the Histokinette system.

During dehydration, samples were placed in histology cassettes lined with foam padding and subjected to Histokinette program 3, consisting of 12 sequential steps: seven ethanol dehydration steps with increasing concentrations, three xylene steps for alcohol removal, and two paraffin infiltration steps. For embedding, cassettes were transferred to a heated embedding chamber. Tissue fragments were removed from the cassettes, oriented with the flat surface facing downward, embedded in paraffin molds, and cooled on a –50 °C plate to solidify the blocks. Paraffin blocks were sectioned into 5 μm-thick slices using a microtome, and slides were incubated at 40 °C for 24 hours to dry. Two representative slides per sample were selected for staining. To account for potential compositional heterogeneity along the thrombus length, proximal and distal fragment ends together with unused slices were included in the histological specimens.

Two staining protocols were used for compositional characterization. Martius-Scarlet-Blue (MSB) staining highlighted RBCs in yellow, fibrin in red, WBCs in dark purple, and connective tissue in blue, whereas CD42b⁺ immunohistochemistry identified platelets in brown and WBCs in purple, leaving RBCs and fibrin in white-gray. Stained slides were digitized using a Nanozoomer scanner at 40× magnification (0.23 μm/pixel resolution) and stored as .ndpi files.

Quantification of thrombus composition was performed on digital images using Orbit Image Analysis. Slides containing artefacts due to sample preparation or scanning were manually excluded. Classification models for MSB and CD42b⁺ staining were trained using supervised machine learning, where representative regions of interest (ROIs) were manually annotated (*Fig. S.6a*). For MSB staining, ROIs included RBCs, white blood cells (WBCs), and fibrin/platelet (FP) aggregates, annotated on five randomly selected training images. For CD42b⁺ staining, ROIs focused on platelet-rich areas.

*Fig. S.6 –* ***Histology stains for thrombus samples and automatic thrombus composition classification and quantification****:* a) example of MSB and CD42b+ stained mechanically tested thrombus sample. Fibrin marked with triangle, RBCs marked with circle, WBCs marked with square, platelets marked with hexagon; b) example of automatic classification and quantification for a MSB-stained slide: after removing the background, the classification model automatically detects and quantifies RBCs, FP and WBCs regions.

To evaluate intra-user variability, three independent classification models were trained and applied to the entire dataset. Each model performed pixel-level classification to segment RBCs, FP aggregates, WBCs, and platelets (*Fig. S.6b*). The outputs of the three models were averaged to obtain final percentage values for each thrombus component. Quantification results were reviewed and validated by an experienced pathologist blinded to clinical and interventional data.

Quantitative histological analysis was performed on 651 MSB-stained sections to assess RBCs, FP aggregates, and WBCs. Thrombi were predominantly composed of FP aggregates (median 69.21%; IQR 40.38%), followed by RBCs (median 28.50%; IQR 40.32%) and WBCs (median 1.43%; IQR 1.21%). Mean composition values were 33.25% RBCs, 64.78% FP, and 1.67% WBCs. Shapiro–Wilk tests indicated that all component distributions significantly deviated from normality (p < 0.001). RBC content ranged from 0.26% to 94.75%, highlighting substantial heterogeneity in thrombus architecture (*Fig. S.7a*).

A total of 642 CD42b⁺-stained sections were analyzed to quantify platelet content. Platelets represented the predominant component (median 51.0%; IQR 33.4%), with values ranging from 0.10% to 97.40%. The remaining composition consisted of non-platelet components (median 48.7%; IQR 33.5%) and a small proportion of WBCs (median 0.20%; IQR 0.40%). Mean values were 51.1% platelets, 0.4% WBCs, and 48.5% other components. Shapiro–Wilk tests again confirmed non-normal distributions for all variables (p < 0.001). These findings highlight the marked variability in platelet content among AIS thrombi (*Fig. S.7b*).

*Fig. S.7 –* ***Quantitative assessment of thrombus composition from MSB- and CD42b^+^ stained histological sections.*** *(a)* MSB-stained sections (n = 651) showing relative content of red blood cells (RBCs, blue), fibrin/platelet aggregates (FP, red), and white blood cells (WBCs, yellow), ordered by decreasing RBC content.
*(b)* CD42b⁺ immunohistochemistry images (n = 642) showing proportions of platelets (blue), WBCs (red), and other components (yellow), matched to the MSB samples for direct comparison. Both panels illustrate marked inter-sample variability in thrombus composition across the cohort.

To evaluate compositional variability across samples and patients, MSB-based composition percentages were analyzed. *Fig. S.8a* shows the average composition per patient, with the number of samples analyzed indicated above each bar. Intra-patient variability was assessed using the coefficient of variation (CV) across samples from the same patient (*Fig. S.8b*), whereas inter-patient variability was calculated using the CV across patient-averaged compositions (*Fig. S.8c*). Inter-patient CV values were 64.5% for RBCs, 28.7% for FP aggregates, and 46.8% for WBCs, confirming the heterogeneous nature of AIS thrombi.

*Fig. S.8 –* ***Per patient thrombus composition****:* a) average composition percentage of all samples from each patient (number of samples per patient reported in black at the top) computed by MSB staining images. RBCs (blue), fibrin/platelets aggregates (red), WBCs (yellow). Intra-patient b) and inter-patient c) coefficient of variation for each thrombus component.

To explore whether thrombus composition differed by occlusion location, univariable linear mixed-effects regression (LMR) models were computed (*Fig. S.9*). No statistically significant differences were observed in RBC, FP, or platelet content among ICA, M1, and M2 occlusions.

*Fig. S.9 –* ***Results for univariable LMR for thrombus composition among occlusion location****:* no statistically significant differences were observed for RBCs, FP, and platelets, among the intracranial carotid artery (ICA), M1 or M2 segments of the middle cerebral artery.

**S.4 Radiological Characterization of Acute Ischemic Stroke Thrombi**

Pre-interventional thin-slice (≤3 mm) non-contrast CT (NCCT) and CT angiography (CTA) scans were analyzed following the previously described imaging protocol.^4^ The first step consisted of automatic rigid registration of NCCT and CTA scans using Elastix ([www.elastix.lumc.nl](http://www.elastix.lumc.nl)). When automatic registration was suboptimal, manual rigid corrections were performed using ITK-SNAP ([www.itksnap.org](http://www.itksnap.org)). Scans with uncorrectable registration errors, incomplete field of view, or undetectable thrombus borders were excluded from analysis.

Radiological thrombus characteristics included thrombus length, thrombus density on NCCT, thrombus perviousness (CTA density minus NCCT density), and the distance from the ICA-terminus to the thrombus (DT). Measurements were performed by a trained observer, with difficult cases reviewed by a second trained observer and verified by a neurointerventional radiologist with 10 years of experience.

To obtain these measurements, up to nine markers were placed along the occluded artery on the registered NCCT and CTA scans using ITK-SNAP (*Fig. S.10*). Marker placement was performed on the axial view, with coronal and sagittal views used for guidance. Three markers were positioned within the thrombus (proximal, middle, and distal regions), while two markers defined the proximal and distal thrombus borders. One marker identified the ICA terminus, and up to three additional markers were placed along the vessel segment between the ICA terminus and the proximal thrombus border.

Thrombus location was determined using the hyperdense artery sign on NCCT (when present) and contrast-filling defects on CTA. If the proximal or distal thrombus boundary could not be clearly identified on CTA, thrombus length was estimated using the hyperdense artery sign on the registered NCCT. When a thrombus extended into two arterial branches, the distal marker was placed in the branch corresponding to the longest thrombus segment.

The three markers placed within the thrombus were used to generate spherical regions of interest (ROIs) with a 1 mm radius. Mean NCCT thrombus density and CTA thrombus density were calculated as the average values across these three ROIs. Thrombus perviousness was computed as the difference between CTA and NCCT densities.

Thrombus length was calculated as the distance between the proximal and distal thrombus border markers, while DT was defined as the path length from the ICA terminus to the proximal thrombus border along the vessel centerline.

*Fig. S.10 –* ***Schematic of marker placement and representative patient example in two imaging views:***
a) schematic illustration of marker placement in coronal (left) and axial (right) views. The red marker indicates the location of the ICA terminus (ICA-T); in cases of a curved vessel extending up to the proximal thrombus border, up to three optional green markers may be placed. Three blue markers indicate the proximal, middle, and distal segments of the thrombus; a yellow marker indicates the proximal thrombus border; and a turquoise marker indicates the distal thrombus border. b) Representative patient example showing marker placement on NCCT and CTA in coronal (left) and sagittal (right) views. Occlusion location is indicated by green boxes.

**S.5 Additional Statistical Analyses**

The raw data used for the GLMR analyses reported in the main manuscript are showed as descriptive statistics in *Fig. S.11*. These data are presented to allow visual comparison between the observed patient-level outcomes and the associations estimated by the GLMR, demonstrating a similar overall pattern and supporting the consistency of the model-based findings.

***Fig. S.11 – Patient-level association between thrombus stiffness and mFPR per occlusion location***: raw patient-level data showing positive (mFPR = 1) and negative (mFPR = 0) outcomes according to thrombus stiffness. Bold regression lines and shaded areas represent the linear regression fits and corresponding 95% confidence intervals, respectively, for each occlusion location, based on patient-level thrombus stiffness values. ICA: intracranial carotid artery; M1, M2: middle cerebral artery; mFPR: first-pass reperfusion (eTICI ≥2b).

To complement the primary analyses based on modified first-pass reperfusion (mFPR; eTICI ≥2b), additional GLMR analyses were performed using first-pass reperfusion (FPR; eTICI ≥2c–3) as the outcome variable (*Fig. S.12*). FPR represents the strictest definition of first-pass success and is commonly used as a procedural outcome metric in EVT studies.

***Fig. S.12 – Influence of thrombus stiffness on FPR probability per occlusion location*:** results from multivariable GLMR adjusted for age, sex, first-line EVT method, and thrombus histological composition. Bold regression lines and solid 95% CI represent the stiffness range derived from the full model accounting for clustering of multiple thrombi per patient. Semi-transparent confidence bands correspond to stiffness ranges obtained using patient-averaged stiffness values. ICA: intracranial carotid artery; M1, M2: middle cerebral artery; FPR: first-pass reperfusion (eTICI ≥2c–3).

The GLMR analysis evaluating the association between thrombus stiffness and FPR yielded results consistent with those obtained for mFPR, with similar trends across occlusion locations. In particular, M1 occlusions showed comparable success probabilities under both outcome definitions, whereas ICA and M2 occlusions exhibited lower first-pass success probabilities, reflecting the greater procedural complexity of thrombectomy in these vascular territories.

These additional analyses support the use of mFPR as the primary outcome metric, as it provided improved stratification of EVT success across occlusion sites while maintaining consistency with results obtained using the stricter FPR definition.

Additional univariable linear mixed-effects regression (LMR) analyses exploring the association between thrombus stiffness and intravenous thrombolysis (IVT) administration, number of EVT attempts, occurrence of procedural complications are illustrated in *Fig. S.13*.

Thrombus stiffness did not differ between patients receiving IVT prior to EVT (either at a peripheral hospital or at the stroke centre) and those without IVT, with marginal stiffness estimates of 0.562 kPa (95% CI: 0.324–0.977) and 0.546 kPa (95% CI: 0.327–0.908), respectively.

Patients experiencing procedural complications during EVT exhibited significantly softer thrombi, with marginal stiffness estimates of 0.257 kPa (95% CI: 0.119–0.555) compared with 0.665 kPa (95% CI: 0.457–0.966) in patients without complications.

No differences in thrombus stiffness were observed among patients requiring different numbers of EVT attempts for recanalization, with overlapping marginal stiffness estimates across attempt groups. Similarly, thrombus stiffness was not associated with the duration of the first EVT attempt, showing a negligible estimated trend (β = -1.7×10⁻⁴, 95% CI: -0.007–0.006).

*Fig. S.13 –* ***Associations between thrombus stiffness and procedural indicators:*** (a) IVT administration; (b) number of EVT attempts required for recanalization; (c) occurrence of procedural complications during EVT. Results are shown as estimated marginal stiffness values with 95% confidence intervals from univariable linear mixed-effects regression models accounting for clustering of multiple thrombus samples within patients.

# References

1.	Goyal M, Menon BK, Van Zwam WH, et al. Endovascular thrombectomy after large-vessel ischaemic stroke: A meta-analysis of individual patient data from five randomised trials. *The Lancet* 2016;387:1723–31.

2.	Jansen IGH, Mulder MJHL, Goldhoorn RJB. Endovascular treatment for acute ischaemic stroke in routine clinical practice: Prospective, observational cohort study (MR CLEAN Registry). *BMJ (Online)* 2018;360.

3.	Cahalane RME, de Vries JJ, de Maat MPM, et al. Tensile and Compressive Mechanical Behaviour of Human Blood Clot Analogues. *Ann Biomed Eng* 2023;51:1759–68.

4.	Santos EMM, Arrarte Terreros N, Kappelhof M, et al. Associations of thrombus perviousness derived from entire thrombus segmentation with functional outcome in patients with acute ischemic stroke. *J Biomech* 2021;128.
